# Comparative Effectiveness of SGLT2i and GLP-1RA on Blood Pressure in Hypertensive Patients with Type 2 Diabetes: A Saudi Multicenter Retrospective Study

**DOI:** 10.3390/jcm14207428

**Published:** 2025-10-21

**Authors:** Ghadah Alshehri, Raghad Alrashidi, Renad Alhaqbani, Reema Almeshari, Nader Bin Sheraim, Alwaleed Alharbi, Majed S. Al Yami, Abdulmohsen Alanazi, Nourah Alsalamah, Amani Alrossies

**Affiliations:** 1 Department of Pharmacy Practice, College of Pharmacy, Princess Nourah Bint Abdulrahman University, Riyadh 11671, Saudi Arabia; 2 College of Pharmacy, Princess Nourah Bint Abdulrahman University, Riyadh 11564, Saudi Arabiarenadhaqbani@gmail.com (R.A.); r.hf.almeshari@gmail.com (R.A.); 3 Clinical Pharmacy Department, King Abdullah Bin Abdulaziz University Hospital, Riyadh 11564, Saudi Arabia; 4 Clinical Pharmacy Department, King Fahad Medical City, Riyadh 12231, Saudi Arabia; 5 Department of Pharmacy Practice, College of Pharmacy, King Saud Bin Abdulaziz University for Health Sciences, Riyadh 11481, Saudi Arabia; 6 Pharmacy Department, King Saud Hospital Unaizah, Unaizah 56437, Saudi Arabia

**Keywords:** sodium–glucose transporter 2 inhibitors, glucagon-like peptide-1 receptor agonists, hypertension, type 2 diabetes, Saudi Arabia

## Abstract

**Background/Objectives:** Sodium–glucose cotransporter-2 inhibitors (SGLT2i) and glucagon-like peptide-1 receptor agonists (GLP-1RA) have shown blood pressure (BP) reduction in type 2 diabetes (T2D). However, head-to-head comparisons in hypertensive patients remain limited. This study assessed the effects of SGLT2i and GLP-1RA on systolic BP (SBP), diastolic BP (DBP), antihypertensive regimen modifications, and adverse events in Saudi patients with both conditions. **Methods:** A retrospective cohort study was conducted between January 2022 and April 2024 using records from two hospitals. Adults with T2D and hypertension who initiated SGLT2i or GLP-1RA and had ≥2 BP readings were included. BP changes were analyzed with ANOVA; adverse events and treatment discontinuation were assessed with Chi-square and Kaplan–Meier analysis. **Results:** Of 505 patients, 291 (57.6%) received SGLT2i and 214 received GLP-1RA. Both classes significantly reduced SBP (*p* < 0.001), and DBP decreased significantly only in the SGLT2i group (*p* < 0.001). Antihypertensive regimen reduction occurred in 6.9% of patients, most commonly among SGLT2i users (74.3%), while 76.8% remained on the same regimen; the remaining patients had other modifications such as dosage adjustments or changes in individual agents. Adverse events occurred in 6.3% of patients with no group differences. Therapy discontinuation was higher with GLP-1RA (12.6%) versus SGLT2i (2.4%, *p* < 0.001). **Conclusions:** Both SGLT2i and GLP-1RA might be considered in patients with T2D and hypertension, with SGLT2i potentially offering additional benefits for DBP reduction and simplifying antihypertensive regimens, which could support clinical decision-making in real-world practice.

## 1. Introduction

Type 2 diabetes (T2D) is one of the fastest growing non-communicable diseases, affecting 529 million adults in 2021 and projected to reach 1.31 billion by 2050 [1]. Its growing prevalence has been consistently associated with increased cardiovascular morbidity and mortality, largely through coexisting risk factors such as obesity, dyslipidemia, and, most importantly, hypertension [2,3,4]. Globally, hypertension affects nearly one billion people, with two-thirds residing in developing countries, and its prevalence is nearly double among individuals with T2D relative to non-diabetic populations [5,6,7]. Hypertension is not only the most common comorbidity in patients with T2D but also the most devastating if left unmanaged, as its coexistence with diabetes exacerbates the risk of cardiovascular events and mortality by 41% and 44%, respectively—much higher than the 7% and 9% increases seen with diabetes alone [8,9,10,11].

T2D management has shifted from a glucocentric to a cardiometabolic approach [12]. As a result, selecting antihyperglycemic treatments with established cardiovascular and renal benefits is now an essential component of T2D management [13]. Sodium–glucose cotransporter-2 inhibitors (SGLT2i) and glucagon-like peptide-1 receptor agonists (GLP-1RA) stand out among diabetes medications for their cardiovascular benefits and improved renal outcomes, as evidenced by multiple cardiovascular outcome trials [14]. Evidence indicates that both GLP-1RA and SGLT2i effectively reduce systolic blood pressure (SBP), with variable effects on diastolic blood pressure (DBP), which reduces the incidence of cardiovascular events among this patient population [15,16,17,18,19,20]. Moreover, recent findings indicate that SGLT2i reduce SBP more effectively than GLP-1RA in T2D patients and have shown a notable distinction in their respective impacts on heart failure and stroke risks, which indicates varied cardiovascular profiles [21]. Despite the growing recognition of the cardiovascular benefits associated with SGLT2i and GLP-1RA in previous studies, few have made direct comparisons between these two classes in terms of blood pressure (BP) effects [14,15,16,17,18,19,20,21].

This gap in knowledge is especially crucial in the context of the Saudi Arabian population, which has witnessed a significant increase in T2D prevalence over a 30-year period, soaring from 8.5% in 1992 to 39.5% in 2022 [22]. At the same time, hypertension also constitutes a major public health issue in Saudi Arabia, affecting approximately one-fourth of the Saudi population aged 14 years and above [23]. While recent studies conducted in Saudi Arabia have explored the effects of SGLT2i and GLP-1RA on BP, there is a noticeable lack of research that focuses specifically on the direct impact these drugs have on BP or on direct comparisons between their respective effects [24,25,26,27]. The primary objectives of the study were to evaluate changes in SBP and DBP over time within each class and to determine which class is more effective, while the secondary objectives were to assess modifications in the antihypertensive regimen and the incidence of adverse events following initiation of the two agents. This retrospective cohort study ultimately aimed to address this gap by directly comparing the impact of these medication classes on BP in Saudi patients with T2D and hypertension.

## 2. Materials and Methods

### 2.1. Study Design

A retrospective cohort study was conducted to evaluate the effects of SGLT2i and GLP-1RA on BP control among patients with T2D and hypertension. This analysis was conducted using electronic health records (EHRs) from King Fahad Medical City (KFMC) and King Abdullah bin Abdulaziz University Hospital (KAAUH) in Saudi Arabia. The EHRs provided comprehensive data on patient demographics, clinical history, medication prescriptions, and BP measurements. The study focused on patients who had been prescribed SGLT2i or GLP-1RA between January 2022 and April 2024.

This study was conducted and reported in accordance with the Strengthening the Reporting of Observational Studies in Epidemiology (STROBE) guidelines (see Supplementary Materials, Table S1) [28].

### 2.2. Study Population

The study included Saudi patients aged 18 years and older with documented diagnoses of T2D and hypertension in their EHRs. Antihypertensive regimen modifications were monitored throughout follow-up visits after treatment initiation. Patients were considered to have remained on the same regimen if the antihypertensive drug class, number of agents, or dosages remained unchanged.

Eligible patients must have visited the KFMC and KAAUH outpatient clinics between January 2022 and April 2024 and been treated with SGLT2i or GLP-1RA (injectable form). Patients were required to have at least two BP readings, including a baseline measurement before treatment initiation, and a follow-up reading taken at least one month after the baseline measurement. Patients admitted at the end of the specified period (baseline visit in April 2024) were followed beyond the inclusion period to ensure two BP measurements after baseline. For patients who discontinued SGLT2i or GLP-1RA for more than a month before the inclusion period and then restarted within the inclusion period, their restart date was considered the baseline and was included. For patients who were on one of the agents and later initiated treatment with the other, follow-up was conducted based on the first drug they started, and data collection ended when the second agent was introduced. Inter-visit intervals were defined as 90–180 days with a ±30-day window to account for scheduling variability, in accordance with the routine outpatient follow-up schedule for patients with T2D and hypertension at both hospitals.

Patients were excluded based on the following criteria: a type 1 diabetes diagnosis, undergoing chronic dialysis, treated with SGLT2i or GLP-1RA before the inclusion period and continued the therapy during the inclusion period, treated with a combination of SGLT2i and GLP-1RA at baseline, and those who had fewer than two BP measurements or insufficient documentation, particularly those with missing or inaccessible drug charts during the relevant outpatient visits.

### 2.3. Data Collection

Data were collected and managed using REDCap (Research Electronic Data Capture) version 7.3.6, a secure, web-based platform designed to support standardized data collection in research studies [29]. Extracted variables included patient demographics (age, gender, weight, height, and smoking status), time since T2D and hypertension onset, comorbidities (e.g., stroke, heart failure, myocardial infarction, transient ischemic attack), antihypertensive medication use before and during follow-up, antidiabetic medication use prior to treatment initiation, heart rate, BP measurements (SBP, DBP), and laboratory values (HbA1c, fasting blood glucose, serum creatinine, creatinine clearance, and estimated glomerular filtration rate).

### 2.4. Statistical Analysis

Data were analyzed using IBM SPSS Advanced Statistics, version 27 (IBM Corp., Armonk, NY, USA). Continuous variables were summarized as means with standard deviations or as medians with interquartile ranges and ranges, depending on distribution, while categorical variables were presented as frequencies and percentages. Normality was assessed using the Kolmogorov–Smirnov and Shapiro–Wilk tests. Comparison between two groups was performed using Student’s *t*-test for normally distributed continuous variables and the Mann–Whitney U test for non-normally distributed variables. Chi-square tests were applied for categorical variables. To assess within-group BP changes over time, repeated measures analysis of variance (ANOVA) followed by Bonferroni post hoc (pairwise comparison) adjustment was performed. To assess between-group differences in BP reduction while controlling for potential confounders, an Analysis of Covariance (ANCOVA) was performed. Dependent variable: Follow-up BP. Fixed factor: Treatment group (GLP-1RA vs. SGLT2i). Covariates: Age, Baseline BP, BMI, HbA1c, Metformin, ACE inhibitors, prevalence of heart failure, beta-blocker use, and renal function parameters. Kaplan–Meier survival analysis was used to estimate the time to adverse events and treatment discontinuation. Log-rank tests were used to compare survival curves between different prognostic subgroups. All tests were two-tailed, and a *p*-value < 0.05 was considered statistically significant.

## 3. Results

### 3.1. Demographic and Clinical Characteristics

**Figure 1:**
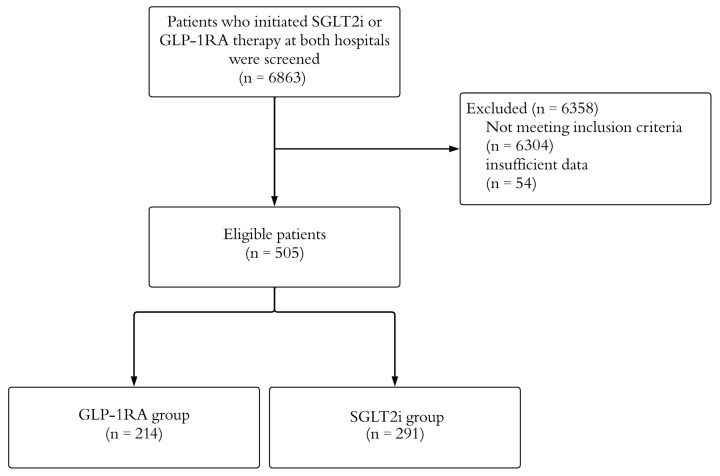
A visual summary of the study flowchart, showing sample size at each filtering step. GLP-1RA: glucagon-like peptide-1 receptor agonists; SGLT2i: sodium–glucose cotransporter-2 inhibitors.

Figure 1 presents the study process flowchart. A total of 6863 patients who had started either SGLT2i or GLP-1RA at both hospitals were screened. After applying the inclusion and exclusion criteria, 505 patients were identified, of whom 291 (57.6%) were treated with SGLT2i and 214 (42.4%) with GLP-1RA. Of the patients in the SGLT2i group, 154 (52.9%) were on dapagliflozin, and 137 (47.1%) were on empagliflozin. In the GLP-1RA group, 164 (76.6%) were on semaglutide, 49 (22.9%) were on liraglutide, and 1 (0.5%) was on dulaglutide. The mean age of the participants was 61.5 ± 10.3 years, 61.4% were female, and 38.6% were male. A significant portion of the participants (86.5%) had a past medical history, with the most common comorbidity being dyslipidemia (81.2%), followed by chronic kidney disease (10.5%). While 5% of the participants had undergone bariatric surgery, 95% had not. Table 1 summarizes the patients’ demographic and clinical characteristics.

### 3.2. SBP Changes

SGLT2i and GLP-1RA both illustrated a statistically significant decrease in SBP between the first and second visits. The reduction in SBP was distinct in the SGLT2i group, which showed a mean decrease from 137.4 ± 17.3 mmHg at baseline to 132.1 ± 16.0 mmHg at the first visit, followed by a further reduction to 130.1 ± 16.4 mmHg at the second visit (*p* < 0.001). Similarly, the GLP-1RA group demonstrated a reduction from 135.4 ± 17.3 mmHg at baseline to 130.4 ± 15.9 mmHg at the first visit and 129.1 ± 14.9 mmHg at the second visit (*p* < 0.001). According to pairwise comparisons, both SGLT2i and GLP-1RA demonstrated a significant reduction in SBP from baseline to the values observed at the first and second visits (*p* < 0.001). However, between the first and second visits, neither group showed significant reductions in SBP (*p* = 0.323 for SGLT2i, *p* = 1.00 for GLP-1RA).

### 3.3. DBP Changes

SGLT2i revealed a statistically significant decrease in DBP over the first and second visits. In the SGLT2i group, DBP at baseline declined from 74.2 ± 11.9 mmHg to 71.9 ± 11.9 mmHg at the first visit and then to 70.9 ± 11.1 mmHg at the second visit (*p* < 0.001). Conversely, the GLP-1RA group showed no statistically significant decline in DBP, with values of 72.1 ± 12.6 mmHg at baseline, 73.1 ± 12.0 mmHg at the first visit, and 71.9 ± 11.8 mmHg at the second visit (*p* = 0.253).

Pairwise comparisons showed that, while the SGLT2i group revealed significant reductions from baseline to the first visit (*p* = 0.001) and from baseline to the second visit (*p* < 0.001), the change between the first and second visits did not reach significance (*p* = 1.00).

### 3.4. Comparison of SGLT2i and GLP-1RA Effects on BP

**Figure 2:**
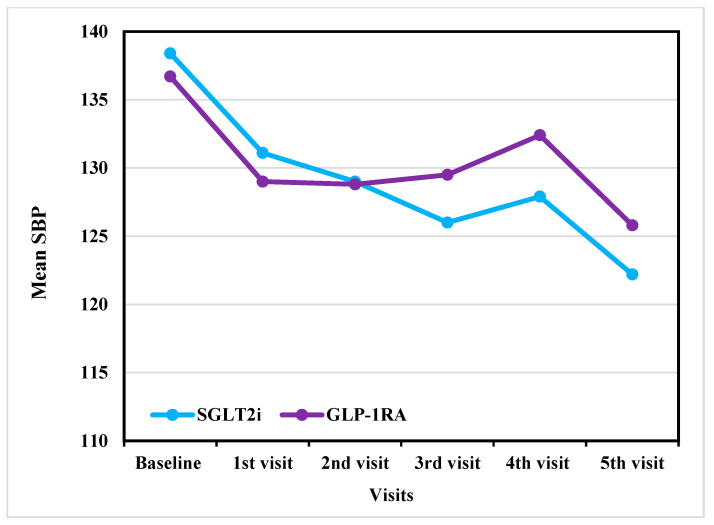
Outcome analysis of SBP reduction across visits in GLP-1RA versus SGLT2i (After adjustment). SBP: systolic blood pressure; GLP-1RA: glucagon-like peptide-1 receptor agonists; SGLT2i: sodium–glucose cotransporter-2 inhibitors.

SGLT2i and GLP-1RA both resulted in moderate reductions in SBP and DBP over time. In SBP, both groups showed a similar gradual decrease from baseline as shown in Figure 2. The SGLT2i group demonstrated a decrease from 137.4 ± 17.3 mmHg at baseline to 121.8 ± 12.4 mmHg at the fifth visit before adjustment. After ANCOVA adjustment, SBP decreased from 138.4 ± 16.6 mmHg at baseline to 122.2 ± 6.6 mmHg at the last visit. While the GLP-1RA group showed a decline from 135.4 ± 17.3 mmHg to 133.0 ± 15.4 mmHg before adjustment. After ANCOVA adjustment, SBP decreased from 136.7 ± 17.7 mmHg at baseline to 125.8 ± 8.1 mmHg over the same period as shown in Table 2.

**Figure 3:**
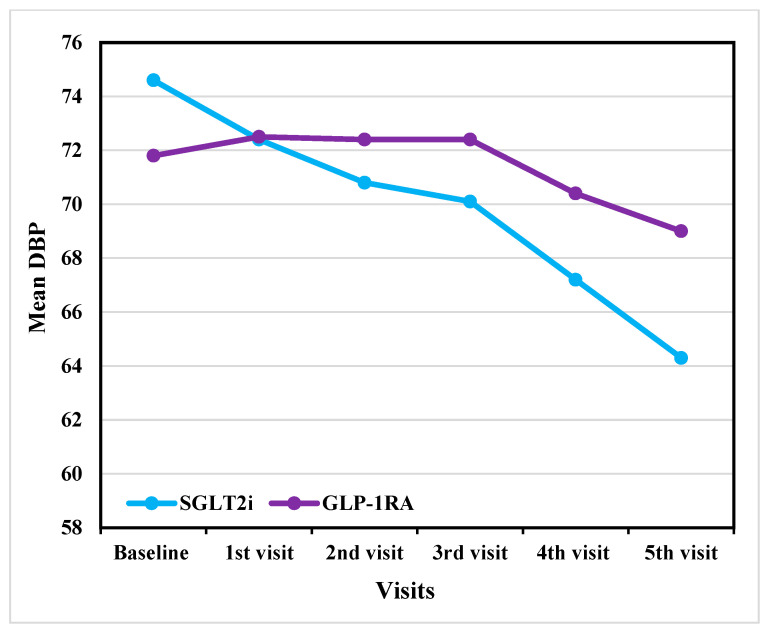
Outcome analysis of DBP reduction across visits in GLP-1RA versus SGLT2i (After adjustment). DBP: diastolic blood pressure; GLP-1RA: glucagon-like peptide-1 receptor agonists; SGLT2i: sodium–glucose cotransporter-2 inhibitors.

In DBP, both groups showed a similar decreasing trend as shown in Figure 3. The SGLT2i group showed a decrease from 74.2 ± 11.9 mmHg at baseline to 67.2 ± 10.1 mmHg at the fifth visit before adjustment. After ANCOVA adjustment, DBP decreased from 74.6 ± 11 mmHg at baseline to 64.3 ± 10.5 mmHg at the last visit, whereas GLP-1RA declined from 72.1 ± 12.6 mmHg to 71.8 ± 9.6 mmHg before adjustment and decreased from 71.8 ± 12.5 mmHg at baseline to 69 ± 9.9 mmHg after ANCOVA adjustment. Despite these trends, none of the differences between the two groups at any time were statistically significant (*p* > 0.05).

### 3.5. Subgroup Analysis

An exploratory subgroup analysis was conducted with the participants who completed all five visits (SGLT2i, n = 23; GLP-1RA, n = 16). A statistically significant decrease in SBP was observed for both agents over the five visits. In the SGLT2i group, a decrease from 133.5 ± 14.3 mmHg at baseline to 121.8 ± 12.4 mmHg at the fifth visit was noted (*p* = 0.017). In the GLP-1RA group, SBP declined from 133.1 ± 13.1 mmHg at baseline to 133.0 ± 15.4 mmHg by the fifth visit (*p* = 0.028).

Based on the results of the pairwise comparison, the SGLT2i group demonstrated a significant reduction in SBP between the baseline and fifth visits (*p* = 0.026). However, no significant differences were observed between the other visits. For the GLP-1RA group, SBP significantly declined between the baseline and second visits (*p* = 0.024) and between the second and fifth visits (*p* = 0.022).

Although the SBP results were significant, neither class showed any statistically significant decline in DBP. For the SGLT2i group, DBP was 67.4 ± 9.4 mmHg at baseline and reached 67.2 ± 10.1 mmHg at the fifth visit (*p* = 0.719). Similarly, the GLP-1RA group’s DBP was 70.9 ± 11.0 mmHg at baseline and reached 71.8 ± 9.6 mmHg at the fifth visit (*p* = 0.702).

### 3.6. Changes in Antihypertensive Medications

To evaluate the impact of the two classes on antihypertensive medication use, we assessed modifications to the antihypertensive regimen following the initiation of either therapy. The antihypertensive medications of 35 patients (6.93%) were discontinued from the baseline, with more discontinuations occurring in the SGLT2i group (n = 26, 74.3%) than in the GLP-1RA group (n = 9, 25.7%). A total of 27 patients (5.34%) increased the number of antihypertensive medications, while 11 (3.76%) switched to a different class of antihypertensive therapy. Of the 431 (85.35%) patients who retained the same number of antihypertensive medications, 29 (6.73%) had their doses increased, while 14 (3.25%) had theirs reduced. Of the entire study population, only one patient had undocumented antihypertensive regimen details. Despite these variations, most of the subjects (n = 388, 76.83%) remained on the same antihypertensive regimen.

### 3.7. Adverse Events and Therapy Discontinuation

Data pertaining to various adverse events associated with either SGLT2i or GLP-1RA were collected. Adverse events were reported in 6.3% of the total study population, with a similar incidence observed in the SGLT2i (n = 18, 6.2%) and GLP-1RA (n = 14, 6.5%) groups. The specific adverse events identified included diabetic ketoacidosis (n = 1, 0.2%), dehydration (n = 1, 0.2%), urinary tract infections (n = 4, 0.8%), gastrointestinal disturbances (n = 16, 3.2%), and hypoglycemia (n = 12, 2.4%). No instances of limb amputation or genital tract infections were reported.

The proportion of patients who remained free from adverse events at 12 months was slightly higher in the SGLT2i group (5.9%) than in the GLP-1RA group (9%), although this difference was not statistically significant (*p* = 0.788). By 18 months, the adverse event rate had risen to 13.5% in the SGLT2i group and 9% in the GLP-1RA group; however, the difference remained statistically insignificant (*p* = 0.788). The overall rates of adverse events remained balanced between the groups over time. Therapy discontinuation was significantly higher in the GLP-1RA group (12.6%) than in the SGLT2i group (2.4%; *p* < 0.001).

## 4. Discussion

This retrospective cohort study compared the BP-lowering effects of SGLT2i and GLP-1RA in Saudi patients with hypertension and T2D, with a focus on changes in SBP, DBP, antihypertensive regimen modifications, and adverse events. Both drug classes significantly reduced SBP; however, only the SGLT2i group achieved a significant reduction in DBP. Notably, most patients maintained BP control without altering their antihypertensive regimens, and adverse event rates were comparable between the groups.

The findings of this study align with previous literature demonstrating the BP-lowering effects of SGLT2i and GLP-1RA in patients with T2D. A systematic review and meta-analysis by Diallo et al. reported an overall reduction in SBP of 2.9 mmHg with SGLT2i and 1.4 mmHg with GLP-1RA [21], reinforcing the greater hypotensive effect of SGLT2i. Similarly, our study observed reductions in SBP in both treatment groups. In the SGLT2i group, SBP decreased from 137.4 mmHg at baseline to 121.8 mmHg at the fifth visit, while the GLP-1RA group showed a decline from 135.4 ± 17.3 mmHg to 133.0 ± 15.4 mmHg over the same period. The observed reductions are clinically relevant, as studies have shown that a 5 mmHg SBP decrease is associated with a 10% reduction in major cardiovascular events [21,30].

Both SGLT2i and GLP-1RA demonstrated a more pronounced effect on SBP in the early treatment phases, with significant reductions observed between the baseline and the first and second visits. However, for DBP, only SGLT2i showed a significant early reduction, whereas GLP-1RA did not exhibit a statistically significant change over the same period. Recent evidence indicates that even modest reductions in DBP are clinically relevant, as they are associated with a decreased risk of stroke and heart disease [31]. This result suggests that SGLT2i has both statistically significant and potentially clinically meaningful benefits.

In an exploratory subgroup analysis of patients who completed all five visits, both SGLT2i and GLP-1RA showed reductions in SBP. However, between-group observations appeared similar for DBP, which differs from the main analysis, where only SGLT2i demonstrated a significant reduction; this discrepancy, based on a small sample size, may reflect limited statistical power rather than true equivalence and should be interpreted with caution. Larger studies with extended follow-up are needed to confirm whether GLP-1RA can achieve comparable effects.

The SBP reduction observed in the SGLT2i group was likely due to natriuresis, osmotic diuresis, and renin–angiotensin–aldosterone system modulation, which leads to early plasma volume reduction and confers long-term vascular benefits [32]. In contrast, GLP-1RA lower SBP through vasodilation and transient natriuresis but have a minimal impact on vascular resistance, which primarily influences DBP [33]. Meanwhile, SGLT2i enhances arterial compliance and provides sustained natriuresis, both of which contribute to DBP reduction [32].

Following treatment initiation with SGLT2i or GLP-1RA, most patients remained on the same antihypertensive regimen, while a smaller subset either had their doses reduced or discontinued one or more antihypertensive agents. The higher rate of antihypertensive medication discontinuation observed in the SGLT2i group occurred alongside significant BP reductions, which might suggest that BP control was maintained even with fewer antihypertensive agents. This finding is consistent with real-world data, as demonstrated by An et al. [34], who observed that initiation of SGLT2i in hypertensive patients led to both significant blood pressure reduction and a reduced requirement for concomitant antihypertensive medications. Taken together, these findings could suggest a potential role for SGLT2i in reducing polypharmacy, a common challenge in patients with T2D and hypertension [35,36]. However, the reasons for antihypertensive medication discontinuation were not explored in this study and may have been influenced by clinician judgment, comorbidities, or perceived patient risk rather than a direct pharmacologic effect. Further research is needed to determine whether this effect could support antihypertensive medication de-escalation in clinical practice, especially among populations at risk of experiencing a medication burden.

Adverse event rates in the two groups were comparable and aligned with findings from the cohort study by Baviera et al. [37]. Therapy discontinuation was significantly higher in the GLP-1RA group (12.6%) than in the SGLT2i group (2.4%), primarily due to gastrointestinal side effects, which accounted for the majority of discontinuations. This finding aligns with the study by Almohaileb et al. [38], which reported that gastrointestinal-related adverse events were among the most common reasons for GLP-1RA discontinuation. In contrast, SGLT2i discontinuation was less frequent and was mainly attributed to dehydration and hypotension. This difference in discontinuation rates suggests that tolerability may play a role in therapy persistence, potentially favoring persistence with SGLT2i.

## 5. Perspectives for Clinical Practice

Our study findings have important clinical considerations. By highlighting the potential role of SGLT2i and GLP-1RA in BP control, our results support their consideration in patients with T2D and hypertension. In clinical practice, these agents are increasingly selected for patients with multiple comorbidities, as they can target several conditions simultaneously. For instance, SGLT2i are widely used in patients with diabetes, heart failure, and chronic kidney disease because of their demonstrated cardiovascular and renal benefits [15,16,19,39], while GLP-1RA are frequently chosen in patients with diabetes and obesity due to their favorable effects on body weight, and metabolic parameters [40].

From a patient-centered perspective, both classes may also contribute to improved quality of life and treatment satisfaction. Semaglutide has been shown to enhance patient-reported quality of life alongside metabolic improvements [40], while dapagliflozin has been associated with greater treatment satisfaction in overweight patients [41]. While these outcomes were not within the scope of this study, evidence from the literature provides complementary insights into the broader therapeutic value of these agents.

Taken together, these considerations suggest that the integration of SGLT2i or GLP-1RA into management decisions for patients with T2D and hypertension reflects a broader clinical approach—prioritizing not only BP reduction but also cardiovascular and renal protection, weight management, and patient-reported outcomes.

## 6. Strength and Limitations

The study provides the first direct comparison of the BP-lowering effects of SGLT2i and GLP-1RA in a real-world Saudi cohort with both hypertension and T2D. The multicenter design and the use of strict inclusion and exclusion criteria strengthen the internal validity of the findings. Moreover, the study is highly relevant to Saudi patients, a population with a particularly high prevalence of both conditions [22,23].

However, several limitations should be acknowledged. The retrospective, non-randomized design restricts the ability to establish a causal relationship between SGLT2i or GLP-1RA use and BP reduction. In addition, potential confounding by indication may have influenced treatment choices, as prescribing decisions could have been shaped by disease severity, comorbidities, or physician preference. This makes it challenging to determine whether the observed BP effects were attributable to the medications themselves or to underlying patient differences. The relatively small sample size in the subgroup analysis further reduced statistical power and limited the generalizability of the results, highlighting the need for larger, prospective studies. Moreover, although outpatient visits were scheduled at 3–6-month intervals, actual visit timing varied across patients, potentially introducing heterogeneity in treatment exposure duration and influencing the magnitude of observed BP changes. Another limitation arises from the retrospective data collection, which precluded detailed information on BP measurement methods and may have introduced variability due to visit-to-visit fluctuations or the white-coat effect. Additionally, data on adherence to the study medications, lifestyle factors (e.g., diet and physical activity), and socioeconomic status were not explored. These areas could be considered for future investigation to provide a more comprehensive understanding of BP control. Future studies should also further investigate the severity and management of clinical consequences to ensure the safe use of these drugs. Finally, as the study was conducted in a specific Saudi population, the generalizability of the findings to other populations may be limited. Future investigations in diverse cohorts with longer follow-up periods are warranted to confirm these results and better evaluate the safety and clinical outcomes of SGLT2i and GLP-1RA in broader clinical contexts.

## 7. Conclusions

In this multicenter, real-world cohort of Saudi adults with T2D and hypertension, both SGLT2i and GLP-1RA were associated with significant SBP reductions, while a significant DBP reduction was observed only with SGLT2i. Most patients remained on the same antihypertensive regimen; de-escalation occurred more often with SGLT2i. Adverse events were comparable between the drug classes, while treatment discontinuation was higher with GLP-1RA. These findings support considering either class for patients with both conditions, with SGLT2i potentially offering added benefit for DBP lowering and regimen simplification. Further prospective studies and randomized trials are warranted to confirm these observations and to define their long-term impact on patient outcomes.

## Figures and Tables

**Table 1:** Baseline characteristics and clinical data of patients treated with SGLT2i and GLP-1RA.

	SGLT2i n = 291	GLP-1RA n = 214	*p*-Value
Demographic characteristics			
Age, Mean ± SD	63.9 ± 9.6	58.3 ± 10.5	<0.001 *
Female sex, n (%)	154 (52.9)	156 (72.9)	<0.001 *
Male sex, n (%)	137 (47.1)	58 (27.1)	<0.001 *
Current smoker	26 (19.4)	17 (12.8)	0.069
Former smoker	26 (19.4)	17 (12.8)	
Non-smoker	82 (61.2)	99 (74.4)	
Heart failure, n (%)	26 (8.9)	9 (4.2)	0.039 *
Myocardial infarction, n (%)	33 (11.3)	18 (8.4)	0.280
Stroke, n (%)	19 (6.5)	13 (6.1)	0.836
TIA, n (%)	1 (0.3)	2 (0.9)	0.577
Time since T2D onset, n (%)			
<1 year	4 (2.0)	8 (5.6)	0.122
1–5 years	35 (17.4)	33 (22.9)	
6–10 years	37 (18.4)	34 (23.6)	
11–15 years	37 (18.4)	21 (14.6)	
16–20 years	27 (13.4)	13 (9.0)	
>20 years	61 (30.3)	35 (24.3)	
Time since hypertension onset, n (%)			
<1 year	6 (8.7)	1 (2.8)	NA
1–5 years	21 (30.4)	11 (30.6)	
6–10 years	14 (20.3)	12 (33.3)	
11–15 years	15 (21.7)	4 (11.1)	
16–20 years	3 (4.3)	4 (11.1)	
>20 years	10 (14.5)	4 (11.1)	
Antihypertensive drugs prior to treatment initiation, n (%)			
ACEi	74 (27.0)	51 (27.9)	0.840
ARB	144 (52.6)	106 (57.9)	0.259
CCB	127 (46.4)	80 (43.7)	0.579
Beta blockers	115 (42.0)	47 (25.7)	<0.001 *
MRA	5 (1.8)	2 (1.1)	0.707
Loop diuretics	37 (13.5)	19 (10.4)	0.319
Thiazide/thiazide-like	74 (27.0)	52 (28.4)	0.741
Alpha blockers	0 (0.0)	1 (0.5)	0.400
Vasodilators	11 (4.0)	12 (6.6)	0.223
Sacubitril/valsartan	5 (1.7)	2 (0.9)	0.704
None	17 (5.8)	31 (14.5)	0.001 *
Antidiabetic drugs prior to treatment initiation, n (%)			
Biguanides	234 (80.4)	177 (82.7)	0.512
Thiazolidinediones	4 (1.4)	1 (0.5)	0.401
Sulfonylureas	113 (38.8)	38 (17.8)	<0.001 *
Dipeptidyl peptidase-4 inhibitors	151 (51.9)	54 (25.2)	<0.001 *
Clinical laboratory data, Mean ± SD			
Weight (kg)	81.1 ± 15.4	95 ± 17.8	<0.001 *
BMI (kg/m^2^)	30.8 ± 5.8	37.2 ± 6.9	<0.001 *
Heart rate (bpm)	81.2 ± 13.9	83.6 ± 13.2	0.059
FBG (mmol/L)	10.1 ± 3.8	9.2 ± 3.8	0.125
HbA1c (%)	8.8 ± 1.8	8.0 ± 1.9	<0.001 *
Serum creatinine (mmol/L)	89.0 ± 40.0	75.9 ± 70.0	0.461
Creatinine clearance (mL/min)	85.3 ± 24.4	96.7 ± 24.8	0.001 *
eGFR (mL/min/1.73 m^2^)	77.5 ± 24.4	92.9 ± 21.5	<0.001 *

Notes: * = statistically significant *p*-value; SGLT2i = sodium–glucose cotransporter-2 inhibitor; GLP-1RA = glucagon-like peptide-1 receptor agonist; T2D, type 2 diabetes; NA = not applicable. TIA, transient ischemic attack; ACEi, angiotensin-converting enzyme inhibitor; ARB, angiotensin II receptor blocker; CCB, calcium channel blocker; MRA, mineralocorticoid receptor antagonist; BMI, body mass index; FBG, fasting blood glucose; HbA1c, hemoglobin A1c; eGFR, estimated glomerular filtration rate.

**Table 2:** Adjusted and unadjusted between-group differences in SBP and DBP reduction (GLP-1RA vs. SGLT2i) using ANCOVA.

SBP	Before Adjustment			After Adjustment		
	SGLT2i	GLP-1RA	*p*-Value	SGLT2i	GLP-1RA	*p*-Value
	Mean ± SD			Mean ± SD		
Baseline	137.4 ± 17.3	135.4 ± 17.3	0.214	138.4 ± 16.6	136.7 ± 17.7	0.525
1st visit	132.1 ± 16	130.4 ± 15.9	0.214	131.1 ± 14.3	129 ± 15.8	0.252
2nd visit	130.1 ± 16.4	129.1 ± 14.9	0.529	129 ± 15.5	128.8 ± 15.1	0.923
3rd visit	129.6 ± 17.7	129.3 ± 13.6	0.888	126 ± 17.1	129.5 ± 15.3	0.072
4th visit	129 ± 14.8	130.6 ± 14.2	0.61	127.9 ± 14.5	132.4 ± 13.4	0.035 *
5th visit	121.8 ± 12.4	133 ± 15.4	0.016 *	122.2 ± 6.6	125.8 ± 8.1	0.264
DBP						
Baseline	74.2 ± 11.9	72.1 ± 12.6	0.064	74.6 ± 11	71.8 ± 12.5	0.060
1st visit	71.9 ± 11.9	73.1 ± 12	0.26	72.4 ± 11	72.5 ± 12	0.167
2nd visit	70.9 ± 11.1	71.9 ± 11.8	0.359	70.8 ± 10.5	72.4 ± 11.6	0.843
3rd visit	71.6 ± 12	71.2 ± 11.3	0.827	70.1 ± 10.1	72.4 ± 11.5	0.860
4th visit	69.7 ± 11.2	69.9 ± 9.5	0.942	67.2 ± 9.9	70.4 ± 9.7	0.879
5th visit	67.2 ± 10.1	71.8 ± 9.6	0.159	64.3 ± 10.5	69 ± 9.9	0.582

Notes: * = statistically significant *p*-value; SGLT2i = sodium–glucose cotransporter-2 inhibitor; GLP-1RA = glucagon-like peptide-1 receptor agonist.

## Data Availability

The original contributions presented in this study are included in the article/Supplementary Material. Further inquiries can be directed to the corresponding author.

## Supplementary Materials

The following supporting information can be downloaded at: https://www.mdpi.com/article/10.3390/jcm14207428/s1, Table S1: STROBE Checklist for Observational Studies.

## Abbreviations

The following abbreviations are used in this manuscript:

ANOVA	Analysis of variance
BP	Blood pressure
DBP	Diastolic blood pressure
EHR	Electronic health record
GLP-1RA	Glucagon-like peptide-1 receptor agonist
HbA1c	Hemoglobin A1c
KFMC	King Fahad Medical City
KAAUH	King Abdullah bin Abdulaziz University Hospital
SBP	Systolic blood pressure
SGLT2i	Sodium–glucose cotransporter-2 inhibitor
T2D	Type 2 diabetes
